# Thoroughly Remold the Localization and Signaling Pathway of TLR22

**DOI:** 10.3389/fimmu.2019.03003

**Published:** 2020-01-17

**Authors:** Jianfei Ji, Zhiwei Liao, Youliang Rao, Wenqian Li, Chunrong Yang, Gailing Yuan, Hao Feng, Zhen Xu, Jianzhong Shao, Jianguo Su

**Affiliations:** ^1^Department of Aquatic Animal Medicine, College of Fisheries, Huazhong Agricultural University, Wuhan, China; ^2^Laboratory for Marine Biology and Biotechnology, Pilot National Laboratory for Marine Science and Technology, Qingdao, China; ^3^State Key Laboratory of Developmental Biology of Freshwater Fish, Changsha, China; ^4^College of Veterinary Medicine, Huazhong Agricultural University, Wuhan, China; ^5^College of Life Sciences, Zhejiang University, Hangzhou, China

**Keywords:** grass carp *(Ctenopharyngodon idella)*, TLR22a, TLR22b, MyD88, IFN, NF-κB, GCRV

## Abstract

TLR22 exists in nearly all the poikilothermic vertebrates and plays a central role in the initiation of innate immunity and activation of adaptive immunity. TLR22 signaling pathway has been characterized in detail in fugu (*Takifugu rubripes*). Here, we thoroughly remold the localization and signaling pathways of TLR22. We characterized TLR22a and TLR22b in grass carp (*Ctenopharyngodon idella*), designated as CiTLR22a and CiTLR22b, and explored the ligand(s), adaptor(s), and signaling pathway(s). Results show that both CiTLR22a and CiTLR22b localize to lysosome, acidic compartment. Correspondingly, CiTLR22a and CiTLR22b directly bind and respond to dsRNA analog poly(I:C) at pH 5, but not at pH 7.4, the physiological pH. Moreover, CiTLR22a and CiTLR22b exhibit antagonistic function in signal transmission, wherein CiTLR22a facilitates the protein and phosphorylation levels of IRF7 and enhances the promoter activities of major IFNs and NF-κBs, while CiTLR22b downregulates IRF7 phosphorylation and IRF3 protein level and suppresses the IFN and NF-κB pathways. Further investigations revealed that CiTLR22a restrains grass carp reovirus (GCRV) replication and protects cells from GCRV infection, whereas CiTLR22b plays a negative role in response to GCRV infection. This is the first time to systematically clarify the signaling pathways of two isotype TLR22s; especially, subcellular localization and adaptor are different from previous TLR22 report, which results from technical limitations. The results will serve the antiviral immune mechanisms in poikilothermic vertebrates and evolutionary immunology.

## Highlights

- The first direct evidence is presented that TLR22 binds dsRNA at acidic pH condition.
- Both TLR22a and TLR22b localize to lysosome and interact with adaptor molecule MyD88, which is thoroughly remolded in poikilothermic vertebrates.
- TLR22a enhances the protein and phosphorylation levels of IRF7, while TLR22b suppresses the protein level of IRF3 and phosphorylation level of IRF7.
- TLR22a and TLR22b antagonize in antiviral immune responses.

## Introduction

TLRs, a major family of pattern recognition receptors, play crucial roles in initiating innate immunity and triggering adaptive immunity by recognizing pathogen-associated molecular patterns (PAMPs) or danger-associated molecular patterns (1, 2). TLRs are type I transmembrane proteins consisting of an extracellular leucine-rich repeat (LRR) domain, a transmembrane domain, and an intracellular Toll/interleukin-1 receptor (TIR) domain (3, 4). The ectodomain functions as a PAMP recognition domain, whereas the endodomain engages in the downstream signaling pathways (5–7). Upon pathogen stimulation, TLRs are activated, initiating signaling cascades that lead to NF-κB and IFN transcription. TLR signaling pathways are roughly classified into two distinct pathways: MyD88-dependent and MyD88-independent (TRIF-dependent) pathways (8). MyD88, the first identified TIR domain-containing adaptor protein, is utilized by all mammalian TLRs, with the exception of TLR3. In Teleost, TLR19 is also found through the MyD88-independent pathway (9). In the MyD88-dependent pathway, MyD88 recruits the members of IL-1 receptor-associated kinase (IRAK) family, in which IRAK and IRAK4 are sequentially phosphorylated to dissociate from the receptor complex, and then associate with TRAF6, while IRAK3 (also called IRAK-M) prevents the progress and acts as a negative regulator (10).

TLRs have been comprehensively investigated in the past decades. So far, 10 and 12 TLRs are identified in human and mice, respectively. At least 21 TLR members have been found in more than a dozen of fish species. However, orthologs of mammalian TLR6 and TLR10 to TLR12 have not been identified in fish. TLRs in teleost have some unique members considered to be fish-specific TLRs, including TLR18 to TLR20 and TLR23 to TLR28 (11). TLR22 occurs in nearly all the poikilothermic vertebrates. In fugu (*Takifugu rubripes*), TLR22 recognizes dsRNA, recruits TICAM-1 (also named as TRIF) to induce IFN, which is considered a functional substitute of TLR3 in mammals and a surveillance molecule detecting dsRNA virus (12, 13). It has been proved that TLR22 responded to poly(I:C) and involved in antiviral immunity in many species, including orange-spotted grouper (*Epinephelus coioides*) (14), large yellow croaker (*Pseudosciaena crocea*) (15). In addition, Chakrapani et al. predicted that the mutation of LRR in TLR22 affects its binding ability with poly(I:C) in *Labeo rohita* (16). These results support that TLR22 has a conservative function in responding to dsRNA among different species.

TLRs localize on the cell surface or inside the cell. The intracellular TLRs, including TLR3, TLR7, TLR8, TLR9, and teleost-specific TLR19, are intrinsically capable of detecting nucleic acid (17). For example, dsRNA of viruses is detected by TLR3 (18) and TLR19, ssRNA of viruses is sensed by TLR7 and TLR8 (19, 20), and dsDNA of viruses is recognized by TLR9 (21). There are several ways in which pathogens enter cells and are sensed by TLRs. Uptake of intact microbes into the endocytic pathway may occur by receptor-mediated endocytosis, phagocytosis, or non-specific fluid phase endocytosis (22). Alternatively, viruses may fuse with the plasma membrane and later be swept into the endosomes either before or during the process of replication as a result of autophagy (17). The autophagy pathway activates type I IFN production in plasmacytoid dendritic cells (DCs) by delivering viral nucleic acids to endosomal TLRs. The viruses by TLR7 recognition require cytosolic viral replication into the lysosome by the process of autophagy (23).

Grass carp reovirus (GCRV), a dsRNA virus, causes severe hemorrhagic disease in juvenile grass carp (*Ctenopharyngodon idella*) and black carp (*Mylopharyngodon piceus*) (24–26). Our previous research demonstrated the important roles of grass carp TLR22 in response to GCRV infection. However, the study mainly focused on genomic organization, single nucleotide polymorphism, and mRNA expression characteristics (27, 28). The ligand, adaptor, localization, and signaling pathways are unknown. Recently, our laboratory reported two TLR22 members in grass carp (*C. idella*), namely, CiTLR22a and CiTLR22b, distributing in different parts of the genome of grass carp and sharing 46% identity in protein sequences (11). This leads to some reflections: what is the difference between CiTLR22a and CiTLR22b in signaling pathways and antiviral immunity? And is this consistent with the previously reported signaling pathway of fgTLR22? In the present study, we found that both CiTLR22a and CiTLR22b are located in lysosome and recognize dsRNA, and then recruit MyD88 to initiate downstream signaling pathways. Further studies found that CiTLR22a is able to inhibit the proliferation of GCRV and plays a positive role against viral infection, while CiTLR22b is capable of promoting the replication of GCRV and exerts the opposite effect in antiviral immunity. The subcellular localization and adaptor are completely different from the traditional views. Better understanding the immune mechanisms serves disease control.

## Materials and Methods

### Cell Culture, Viral Infection, and Reagents

*Ctenopharyngodon idella* kidney (CIK) cells were provided by China Center for Type Culture Collection. They were cultured in DMEM supplemented with 10% FBS (Gibco), 100 units/ml penicillin (Sigma), and 100 units/ml streptomycin (Sigma), in a humidified atmosphere of 5% CO_2_ incubator (Thermo Scientific) at 28°C.

GCRV-097, a type II GCRV strain, was propagated in CIK cells and stored at −80°C. For viral infection, CIK cells were plated for 24 h in advance and then infected with GCRV-097 at a multiplicity of infection of 1.

DiI (Beyotime), PGN (peptidoglycan), ultrapure LPS (L4391), poly(I:C), and IPTG (isopropyl-d-1-thi-ogalactopyranoside) were purchased from Sigma-Aldrich. Biotin-poly(I:C) and streptavidin agarose beads were purchased from Thermo Fisher Scientific. dsDNA was prepared and purified from the cDNA template of CIK cells. Hoechst 33342 was purchased from AAT Bioquest. FuGENE^®^ 6 transfection reagent was purchased from Promega. All the restriction enzymes were purchased from Thermo Scientific. All the primer syntheses and DNA sequencings were carried out in AuGCT biotechnology Co., Ltd., Wuhan, China. We ensured that the experiments followed the ethical guidelines of Huazhong Agricultural University and confirmed that all experimental protocols were approved by Huazhong Agricultural University.

### Plasmid Constructions, RNA Interference, and Transfections

pCMV-eGFP and pCMV-eGFP-CMV-SV40 were employed as original plasmids for the constructions of expression vectors. For the subcellular localization studies, the full-length open reading frames of CiTLR22a (GenBank accession number HQ676542) and CiTLR22b (GenBank accession number KY824797) were amplified from grass carp spleen cDNA with corresponding primers (Supplementary Table 1) and digested with restriction enzymes, then ligated into pCMV-eGFP to construct pTLR22a-eGFP and pTLR22b-eGFP fusion vectors, respectively. With the same method, CiTLR22a-myc, CiTLR22b-myc, MyD88-HA, TRIF-HA, and TIRAP-HA were ligated into pCMV-eGFP-CMV-SV40 to obtain overexpression vectors. Other localization fusion vectors (LAMP2-RFP, RAB5-RFP, RAB7-RFP, MyD88-RFP, TRIF-RFP, and TIRAP-RFP) and luciferase reporter plasmids (pIRF3pro-Luc, pIRF7pro-Luc, pIFN1pro-Luc, pIFN3pro-Luc, pIFNγ2pro-Luc, pNF-κB1pro-Luc, and pNF-κB2pro-Luc) were constructed in our previous studies (9, 29, 30). All the vectors were transfected into CIK cells by FuGENE^®^ 6 Transfection Reagent (Promega) according to the manufacturer's instructions.

To knock down the expression of TLR22a and TLR22b, RNA interference assay was performed by transfecting siRNA targeting TLR22a and TLR22b mRNA. Three siRNA sequences for TLR22a (s1: 5′-UAUAUAAUGUGAUUUGUUGUA-3′, s2: 5′-UCUAAAAUCCGUGUAUUUCUG-3′, s3: 5′-UUUUUGUUAGGUUUAACACCU-3′) and three siRNA sequences for TLR22b (s1: 5′-UAUGUUUUGUGCAUAUUUCAA-3′, s2: 5′-AUAAAACUUUUAAGAUUAGAC-3′, s3: 5′-AUUUACUUUUCUUAAACUGAU-3′) were designed. The silencing effects of the three TLR22a and TLR22b siRNA candidates were evaluated by real-time quantitative RT-PCR (qRT-PCR) as well as a negative control siRNA provided by the supplier.

All the vectors and siRNAs were transfected into CIK cells by FuGENE 6 transfection reagent (Promega) according to the manufacturer's instructions.

### qRT-PCR

Total RNAs were isolated using RNAiso Plus (TaKaRa, Japan), and cDNA syntheses were performed according to a previous report (31). mRNA expressions of VP4 and IRAK-M (GenBank accession number MH590729) were quantified using SYBR Premix Ex Taq II reagent (TaKaRa, Japan) and a LightCycler^®^ 480 II Real-time PCR system (Roche, Switzerland). Primers were listed in Supplementary Table 2. Elongation factor 1α was employed as an internal control gene for cDNA normalization, and the data were analyzed using the 2^−ΔΔCT^ method as described previously (32).

### Luciferase Activity Assays

CIK cells were seeded in 24-well plates at a density of 5 × 10^5^ cells/ml for 24 h and co-transfected with the indicated luciferase reporter plasmid, overexpression plasmid, and control reporter plasmid. pRL-TK vector was used as an internal control to normalize the expression level of the transfected plasmid. At 24 h post-transfection, cells were stimulated with poly(I:C) for 24 h. Then cells were washed with PBS, lysed with Passive Lysis Buffer (Promega), and assayed for luciferase activities in a luminometer by the Dual-Luciferase Reporter Assay System (Promega). The luciferase reading of each sample was first normalized against that in the pRL-TK level, and the relative light unit was presented as the ratio of firefly luciferase to renilla luciferase. The results were obtained from four independent experiments, and each was performed in triplicate.

### Antiviral Activity Assays

To evaluate the antiviral activities of CiTLR22a and CiTLR22b, CIK cells were transfected with CiTLR22a-myc, CiTLR22b-myc TLR22a siRNA, and TLR22b siRNA for 24 h and infected with GCRV at a multiplicity of infection of 1. At 48 h post-challenge, cells were collected for virus quantification. mRNA expression of VP4 was examined by qRT-PCR, and protein level of VP56 was detected by Western blotting (WB).

For viral titer assay, supernatants at 24 h post-GCRV infection were serially diluted in 10-fold and incubated with CIK cells in a 96-well plate to determine TCID_50_ (50% tissue culture infective dose). Cells were incubated at 28°C for 7 days. On day 7, the plates were examined for the presence of viral cytopathic effect (CPE) under the microscope.

For standard plaque assay, cells overexpressed CiTLR22a, CiTLR22b, or vector were severally seeded in 24-well plates (5 × 10^5^ cells/well) overnight, then infected with 2-fold-diluted GCRV at indicated densities. After 48 h post-infection, cells were fixed with 10% paraformaldehyde for 10 min at room temperature and stained with 0.05% (wt/vol) crystal violet (Sigma) for 30 min, then washed with water and drained. Subsequently, the plates were photographed under a light box (Bio-Rad).

### Abs, Co-immunoprecipitation, and WB

Anti-IRF7 rabbit polyclonal antiserum and anti-VP56 mouse polyclonal antiserum were previously prepared in our laboratory (9, 29). Anti-IRF3 rabbit polyclonal antiserum was prepared and presented by Prof. Yibing Zhang, Institute of Hydrobiology, Chinese Academy of Sciences, Wuhan, China (33). Anti-HA tag primary mouse monoclonal Ab (ab18181), anti-myc tag primary rabbit polyclonal Ab (ab9106), anti-GST tag primary mouse monoclonal Ab (ab36415), and anti-β-tubulin primary rabbit polyclonal Ab (ab6046) were purchased from Abcam. IRDye 800CW donkey anti-rabbit IgG (H + L) (P/N 925-32213) and anti-mouse IgG (H + L) (P/N 925-32212) secondary Abs were purchased from LI-COR. Goat-anti-mouse Ig-HRP conjugate secondary Ab (A0216) was purchased from Beyotime.

To determine whether CiTLR22a and CiTLR22b interact with the potential adaptor, co-immunoprecipitation (CoIP) experiment was performed as previously described (9). Briefly, CIK cells in 10-cm^2^ dishes were co-transfected with the corresponding vectors for 24h. The cells were lysed in immunoprecipitation lysis buffer for 30 min on ice, and the cellular debris was removed by centrifugation at 12,000 × g for 30 min at 4°C. The supernatant was transferred to a fresh tube and incubated with 1 μg of Ab with gentle shaking overnight at 4°C. Protein A + G sepharose beads (30 μl) (Beyotime) were added to the mixture and incubated for 2 h at 4°C. After centrifugation at 3,000 × g for 5 min, the beads were collected and washed four times with lysis buffer. Subsequently, the beads were suspended in 20 μl 2 × SDS loading buffer and denatured at 95°C for 10 min, followed by WB detection.

For WB analysis, protein extracts were separated by 8–12% SDS-PAGE gels and transferred onto nitrocellulose membranes (Millipore). The membranes were blocked in fresh 3% non-fat dry milk dissolved in TBST buffer at 4°C overnight, then incubated with appropriate primary Abs for 2 h at room temperature: anti-HA (1:1,000), anti-myc (1:1,000), anti-β-Tubulin (1:5,000), anti-IRF3 (1:1,000), anti-IRF7 (1:1,000), and anti-VP56 (1:1,000), respectively. Then they were washed three times with TBST buffer and incubated with secondary Ab for 1 h at room temperature. After washing three times with TBST buffer, the nitrocellulose membranes were scanned and imaged by an Odyssey^®^ CLx Imaging System (LI-COR). The results were obtained from three independent experiments.

### LRR Recombinant Expression, Purification, and PAMP-Binding Activities

The fragments encoding the extracellular region (LRR domain) of CiTLR22a and CiTLR22b were obtained by PCR amplification with primers listed in Supplementary Table 1. Afterward, the purified PCR products were digested with corresponding restriction enzymes and then ligated into the pGEX-4T1 expression vector. Recombinant proteins were expressed in *Escherichia coli* BL21 cells, denatured in 8 M urea, and purified by an affinity chromatography with GST-resin (GenScript, Nanjing, China) according to the manufacturer's instructions.

For PAMP-binding assay, the activities of LRR protein binding PAMPs [LPS, PGN (peptidoglycan), dsDNA, poly(I:C)] were measured by ELISA method as previously described and revised by ourselves (9). Briefly, 96-well microtiter plates were coated with PAMPs (10 μg/well), washed with TBST, and blocked with 1% BSA. One hundred microliters of several concentrations of LRR proteins was added to the corresponding wells. The protein expressed by empty vector was employed as a control. After incubation at 18°C for 3 h, the plates were washed three times with PBS with tween 20 (PBST), and 100 μl of anti-GST (1:1,000) was added as the first Ab. After incubation at 37°C for 1 h, the plates were washed and incubated with 100 μl of goat-anti-mouse Ig-HRP conjugate (1:3,000) as the second Ab for 1 h. The plates were washed four times with TBST for 5 min, and 100 μl of 3,3′,5,5′-tetramethylbenzidine solution (PA107, Tiangen, China) was added to each well and then incubated at room temperature in the dark for 15 min. The reaction was stopped by adding 50 μl of 2 M H_2_SO_4_. The absorbance was measured on Synergy™ 4 Hybrid Microplate Reader (BioTek, Winooski, VT, USA) at 450 nm. The wells filled with 100 μl TBS were used as a negative control. The examination was performed by three independent experiments.

### poly(I:C) Pull-Down Assay

poly(I:C) pull-down assay was performed as previously described (34) with minor modifications. CIK cells overexpressed with CiTLR22a-myc or CiTLR22b-myc were lysed in buffer [50 mM Tris-Cl, 150 mM NaCl, 5 mM EDTA, 1% IGEPAL CA-630 (pH 5), and Roche complete protease inhibitor cocktail] or buffer 50 mM Tris-Cl, 150 mM NaCl, 5 mM EDTA, 1% IGEPAL CA-630 (pH 7.4), and Roche complete protease inhibitor cocktail. Lysates were incubated with biotin-poly(I:C) for 1 h at room temperature with gentle rotation. Cell lysates were then mixed with streptavidin agarose beads for 2 h at 4°C. The beads were washed three times with lysis buffer and analyzed by immunoblot with anti-myc Ab.

### Confocal Microscopy

CIK cells were co-transfected with the indicated plasmids and plated onto coverslips in 12-well plates for 24 h, then the cells were washed, fixed, and stained as reported previously (9). Finally, images were taken with an UltraVIEW VoX 3D Live Cell Imaging System (PerkinElmer).

### Statistical Analysis

Statistical analysis and presentation graphics were carried out by the SPSS 16.0 and GraphPad Prism 6.0 software. Results were shown as mean ± SD from at least three independent experiments. All data were subjected to one-way ANOVA, followed by an unpaired, two-tailed *t*-test. The *p* value < 0.05 was considered as a statistically significant difference (*p < 0.05, **p < 0.01, ***p < 0.001).

## Results

### CiTLR22a and CiTLR22b Localize to Lysosome

**Figure 1:**
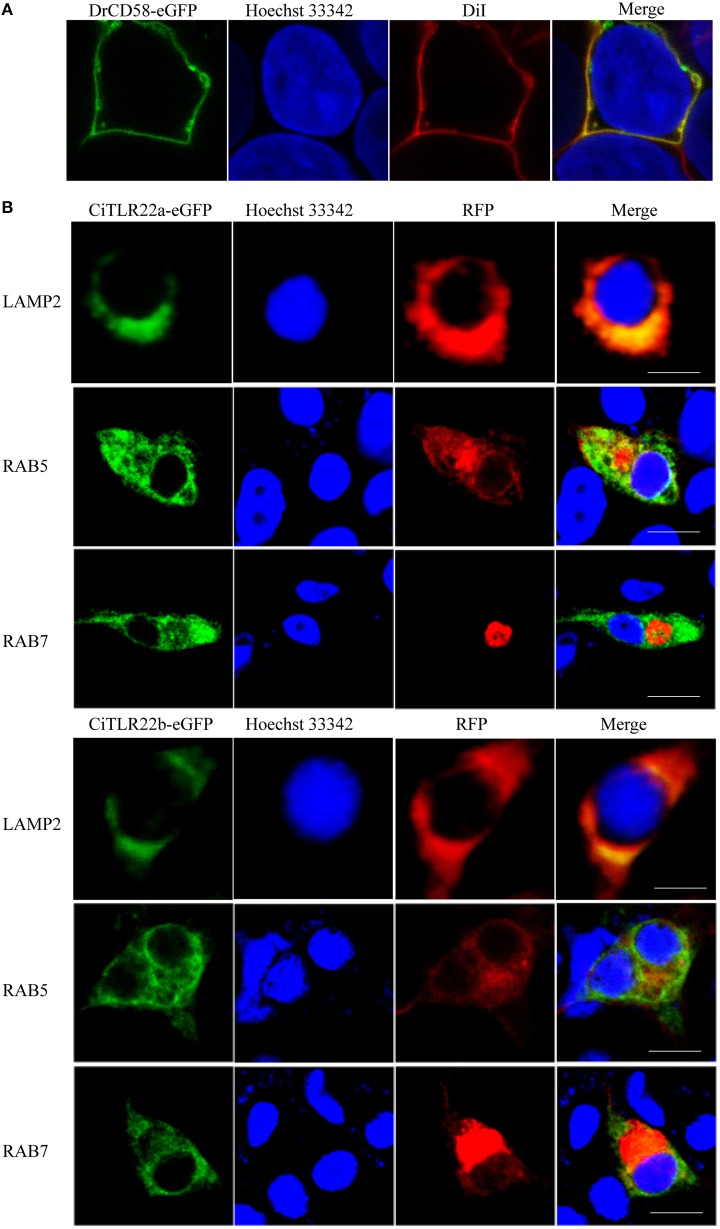
CiTLR22a and CiTLR22b localize to lysosome by colocalization analysis of confocal fluorescence microscopy. **(A)** *Ctenopharyngodon idella* kidney (CIK) cells were transfected with DrCD58-eGFP, then the cell membrane was stained with DiI and cell nucleus was stained with Hoechst 33342. Bar, 10 μm. **(B)** CIK cells were co-transfected with CiTLR22a-eGFP, CiTLR22b-eGFP, and LAMP2-RFP, a lysosome protein marker; RAB5-RFP, an early endosome protein marker; or RAB7-RFP, a late endosome protein marker, respectively, and seeded on microscopic coverglasses in 12-well plates. After 24 h, the cells were fixed with 4% (v/v) paraformaldehyde, stained with Hoechst 33342, and examined by a confocal microscope. Green signals represent overexpressed CiTLR22a and CiTLR22b, and red signals stand for overexpressed LAMP2, RAB5, or RAB7. Blue staining indicates the nucleus. The yellow in the merged images indicates the colocalization between CiTLR22a, CiTLR22b, and lysosome (original magnification ×40; oil immersion objective). All the experiments were repeated at least three times. Bar, 10 μm.

The subcellular localizations of TLRs have a critical impact on their functions in sensing ligands (17). For precise subcellular localization of CiTLR22a and CiTLR22b, confocal microscopy was performed. The primary test showed that both CiTLR22a-eGFP and CiTLR22b-eGFP were detected inside the cells (data not show), while DrCD58-eGFP was localized to cell surface (Figure 1A), which was consistent with a previous report (35), indicating that eGFP tag did not affect the localization of them as DrCD58-eGFP localized to cell surface. To identify the intracellular compartment(s) where CiTLR22a and CiTLR22b enrich, markers of intracellular organelles were used to investigate the colocalization of CiTLR22a and CiTLR22b in different cellular compartments. We engineered CiTLR22a-eGFP and CiTLR22b-eGFP chimeric constructs and co-transfected them with different markers of intracellular organelles in CIK cells. As shown in Figure 1B, CiTLR22a and CiTLR22b colocalize with organelle maker LAMP2 for lysosome, implying that CiTLR22a and CiTLR22b locate at lysosome.

### CiTLR22a and CiTLR22b Recognize dsRNA

**Figure 2:**
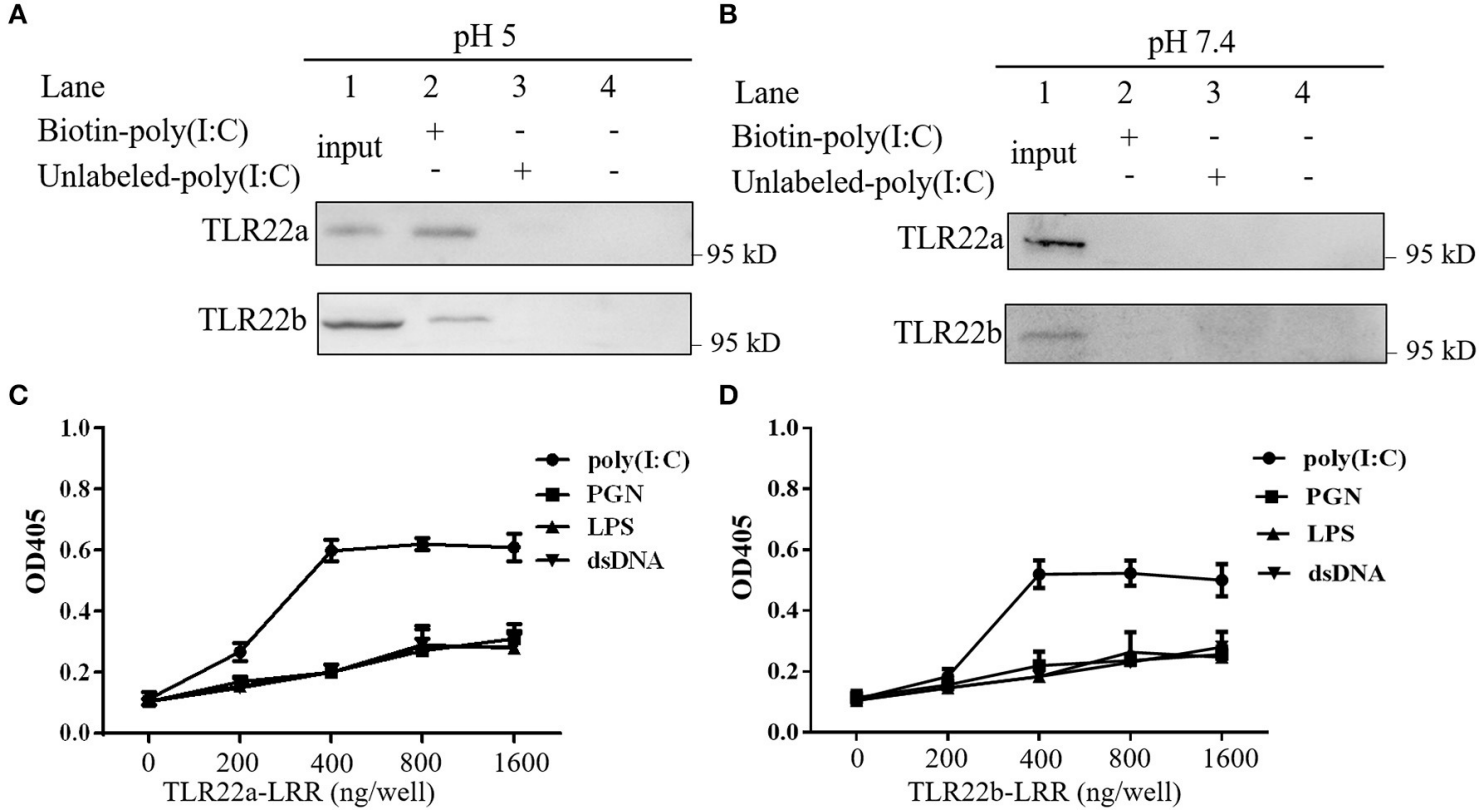
CiTLR22a and CiTLR22b bind dsRNA analog poly(I:C). Cell lysates of overexpressed CiTLR22a and CiTLR22b were incubated with biotin-poly(I:C) (100 ng/ml) or unlabeled poly(I:C) at pH 5.0 **(A)** or pH 7.4 **(B)** for 1 h. Complexes were pulled down by streptavidin beads and analyzed by Western blotting (WB) with anti-myc Ab. **(C,D)** The binding abilities between CiTLR22a-LRR, CiTLR22b-LRR, and different pathogen-associated molecular patterns (PAMPs) were analyzed by ELISA at pH 5.0. Data shown are representative of at least three independent experiments.

A previous study has shown that fgTLR22 recognized dsRNA (13). To detect whether CiTLR22a and CiTLR22b can sense dsRNA, poly(I:C) pull-down and PAMP-binding assays of CiTLR22a, CiTLR22b, and poly(I:C) were performed. As shown in Figures 2A,B, CiTLR22a and CiTLR22b were readily pulled down using biotinylated poly(I:C) as a bait at pH 5, while at pH 7.4, the physiological pH, CiTLR22a, and CiTLR22b were not detectable (Figure 2B), indicating that the interactions require acidic pH. Moreover, The interactions between CiTLR22a-LRR, CiTLR22b-LRR, and PAMPs were observed at pH 5, but not at pH 7.4 (Figures 2C,D; Supplementary Figure 1), indicating that the purified LRR proteins *in vitro* can bind to poly(I:C). All the data above demonstrated that CiTLR22a and CiTLR22b recognize dsRNA occurring in acidic compartments.

### CiTLR22a and CiTLR22b Recruit MyD88 as Adaptor

**Figure 3:**
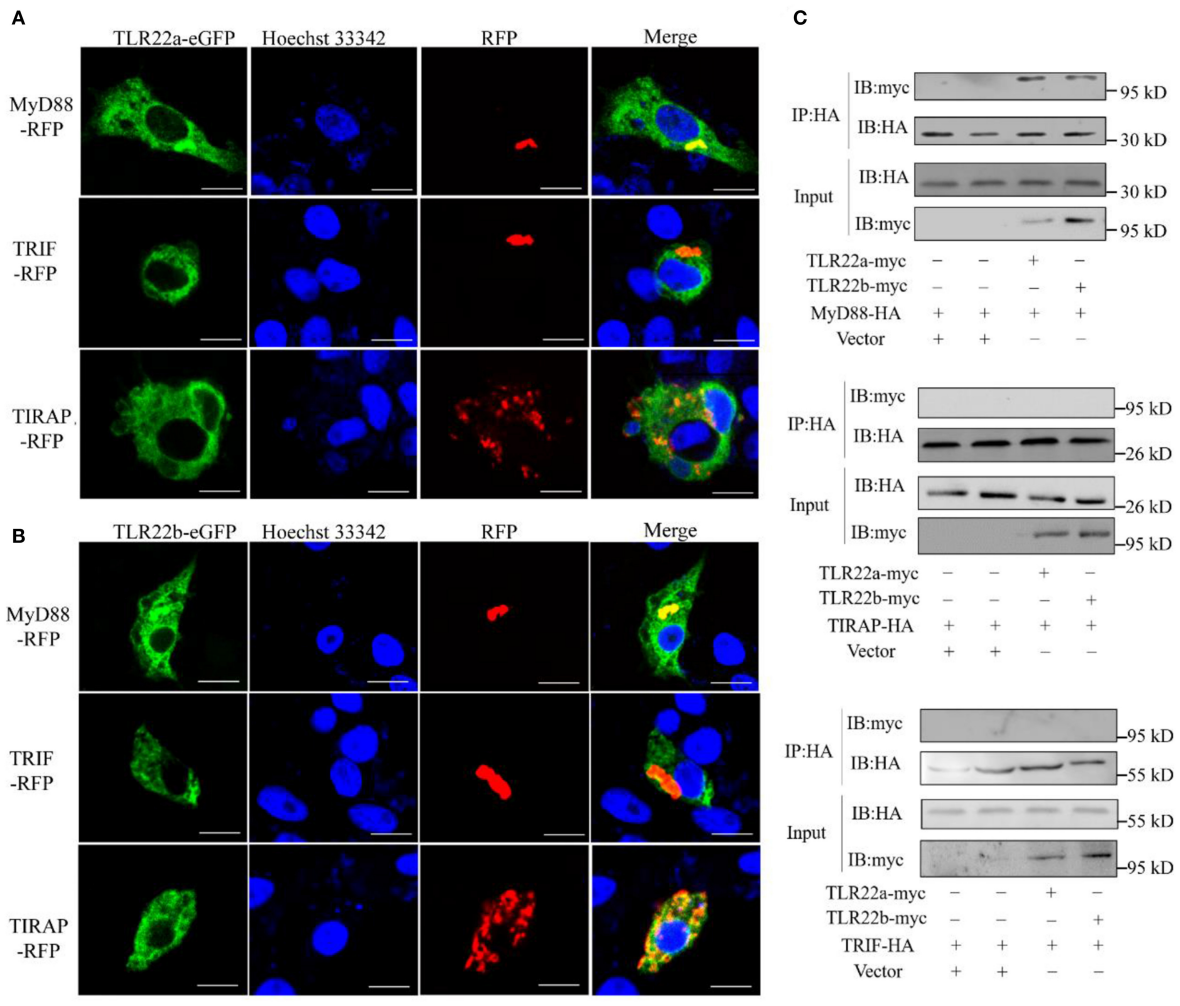
Both CiTLR22a and CiTLR22b colocalize and interact with MyD88 by confocal fluorescence microscopy, co-immunoprecipitation (CoIP) and immunoblot (IB). **(A,B)** CIK cells was co-transfected with CiTLR22a-eGFP, CiTLR22b-eGFP and MyD88-RFP, TRIF-RFP, or TIRAP-RFP as indicated, respectively, and seeded on microscopic coverglasses in 12-well plates. After 24 h, the cells were fixed with 4% (v/v) paraformaldehyde and stained with Hoechst 33342, then subjected to confocal microscopy analysis. Green signals represent overexpressed CiTLR22a and CiTLR22b; red signals stand for overexpressed MyD88, TRIF, or TIRAP; and blue staining indicates the nucleus. The yellow in the merged images indicates colocalization between CiTLR22a, CiTLR22b, and MyD88 (original magnification ×40; oil immersion objective). **(C)** CIK cells in 10-cm^2^ dishes were transfected with the indicated plasmids. After 24 h, CoIP was performed with anti-HA monoclonal antibody. Mouse IgG was used as the control. IB was done with anti-HA and anti-myc Abs, respectively. All the experiments were repeated at least three times. Bar, 10 μm.

CiTLR22a and CiTLR22b bind dsRNA in lysosome. What is (are) the downstream adaptor(s) of CiTLR22a and CiTLR22b? The genome of grass carp encodes five TLR adaptor proteins: MyD88, TIRAP, TRIF, SARM1, and BCAP. TRAM (TRIF-related adaptor molecule), an adaptor of TLRs (8), is not found in teleost and may lose in teleost lineage during evolution. SARM1 and BCAP only serve as negative regulators in TLR signaling (36–38). So, MyD88 (GenBank accession number FJ843088), TRIF (GenBank accession number KC333648), and TIRAP (GenBank accession number KF735057) were chosen as candidate adaptors of CiTLR22a and CiTLR22b. Colocalization was performed by confocal microscopy. The results clearly showed that CiTLR22a and CiTLR22b colocalize with MyD88, no other adaptors (Figures 3A,B). The result indicated that MyD88 is the potential adaptor of CiTLR22a and CiTLR22b. To further test whether CiTLR22a and CiTLR22b utilize MyD88 as an adaptor, myc-labeled CiTLR22a and CiTLR22b and HA-labeled TRIF, MyD88, and TRIAP vectors were constructed. CoIPs were carried out. The results verified the interaction between CiTLR22a, CiTLR22b, and MyD88 (Figure 3C). None of the other interactions were detectable. Both colocalization and CoIP experiments demonstrated that MyD88 is the adaptor of CiTLR22a and CiTLR22b.

**Figure 4:**
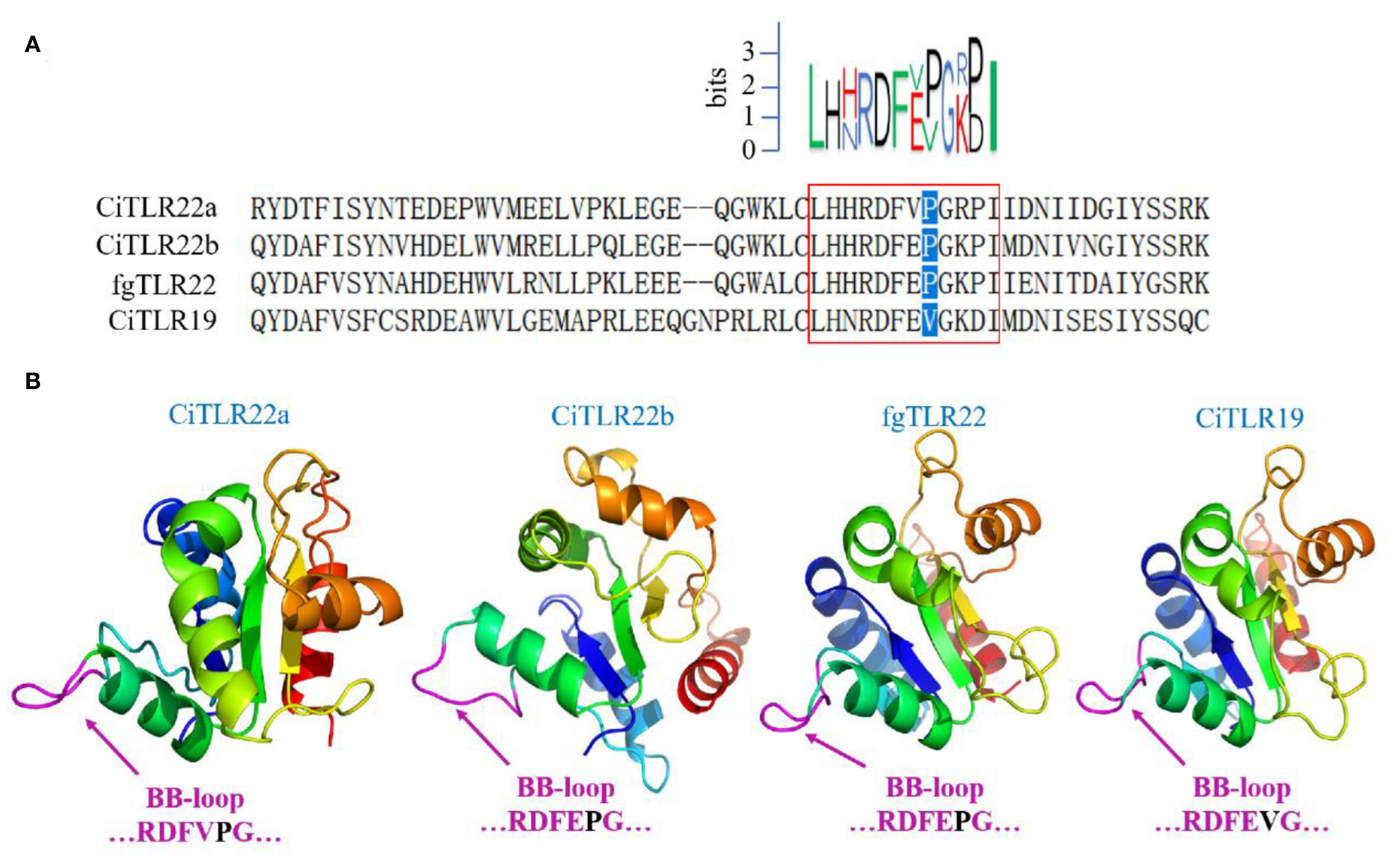
Amino acid sequence alignment and crystal structure of the TLR Toll/interleukin-1 receptor (TIR) domain. **(A)** Sequence logo (top) represents the conserved motif identified by MEME. Sequences in red box are the BB-loop sequences in designated TLRs. The proline residues highlighted in blue determine the adaptor protein bound by TLRs. All TLRs, except CiTLR19, have a proline in the BB-loop. **(B)** The predicted three-dimensional structures were generated using SWISS-MODEL, and figures are generated by PyMol. One hundred percent of the TIR domains of TLRs were modeled with 99.9% confidence by the single highest scoring template as the TIR domain of TLR2. The BB-loop was indicated within the TIR domains.

However, these results above contradict previous research that fgTLR22 utilizes TICAM-1 as an adaptor (13). In the previous studies, the BB-loop of the TIR domain of TLRs was shown to interact with adaptors (39, 40). In addition, all human TLRs, except TLR3, have a proline residue in the BB-loop thought to bind to MyD88 (41, 42). So, to confirm our results above, the TIR amino acid sequences and crystal structure of CiTLR22a, CiTLR22b, fgTLR22, and CiTLR19 were aligned and predicted, respectively. The results showed that all TLRs, except TLR19, which has been proved that its adaptor is TRIF, have a proline in the BB-loop, indicating that MyD88 is the adaptor of CiTLR22a, CiTLR22b, and fgTLR22 (Figures 4A,B).

### CiTLR22a as a Positive and CiTLR22b as a Negative Mediator in IFNs and NF-κB Induction

**Figure 5:**
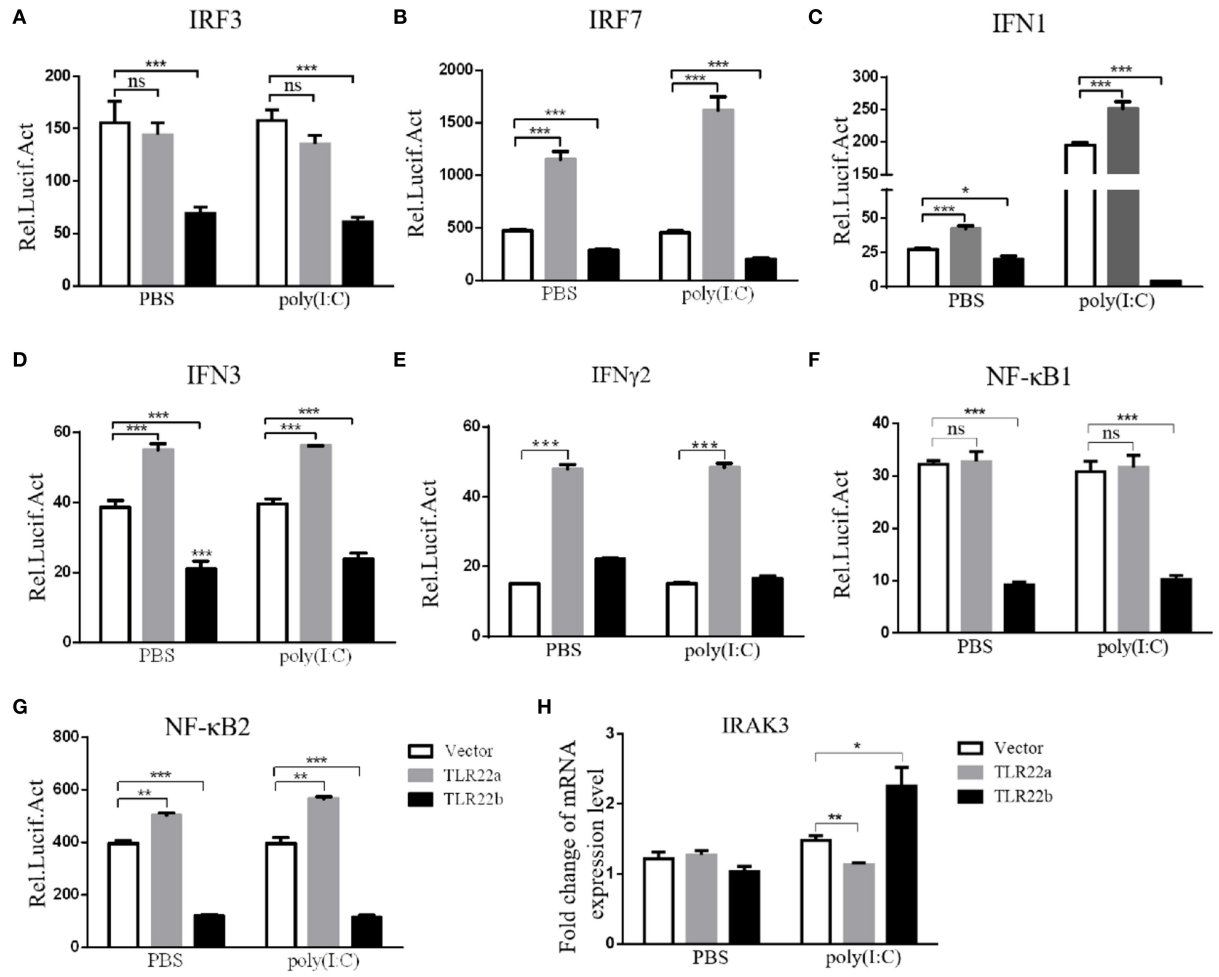
CiTLR22a and CiTLR22b show the opposite effect on IFN and NF-κB pathways by Dual-Luciferase Reporter Assay System and qRT-PCR. **(A–G)** CIK cells were seeded in 24-well plates overnight and co-transfected with 380 ng of pCiTLR22a-myc, pCiTLR22b-myc, or empty vector; 380 ng of each target plasmid (pIRF3pro-Luc, pIRF7pro-Luc, pIFN1pro-Luc, pIFN3pro-Luc, pIFNγ2pro-Luc, pNF-κB1pro-Luc, or pNF-κB2pro-Luc); and 38 ng of the pRL-TK control reporter vector. Twenty-four hours later, the cells were stimulated with poly(I:C) or unstimulated. The luciferase activities were examined at 24 h post-challenge. **(H)** CIK cells were transfected with pCiTLR22a-myc, pCiTLR22b-myc, or empty vector in 12-well plates. Twenty-four hours later, the cells were challenged with poly(I:C) for 24 h. Then the cells were harvested for qRT-PCR to quantify the relative expression levels of IRAK3. All the experiments were repeated in triplicate. *p < 0.05, **p < 0.01, ***p < 0.001.

**Figure 6:**
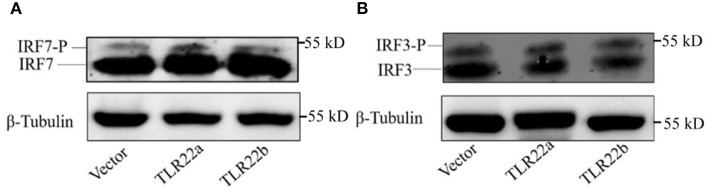
CiTLR22a and CiTLR22b regulate the protein and phosphorylation levels of IRF3 and IRF7 by WB. CIK cells were transfected with pCiTLR22a-myc or pCiTLR22b-myc in 6-well plates for 24 h incubation, then stimulated with poly(I:C) for 24 h. WB was conducted with anti-IRF7 **(A)** and anti-IRF3 antiserums **(B)**, respectively. “-P” indicates the phosphorylation. All the experiments were repeated triplicately.

IFNs and NF-κBs are key important molecules involving in PAMP-triggered immune responses (43, 44). To explore the roles of CiTLR22a and CiTLR22b in dsRNA-mediated IFN and NF-κB induction, luciferase reporter assays were performed to examine the promoter activities of IFNs and NF-κBs upon CiTLR22a and CiTLR22b overexpression. As shown in Figure 5, the promoter activities of IFN1, IFN3, and NF-κB2 genes were significantly increased in CiTLR22a overexpressed cells under MOCK condition, and these trends were more pronounced in the case of poly(I:C)-stimulated condition, whereas the opposite tendency was observed in CiTLR22b overexpressed cells. These results demonstrated that CiTLR22a and CiTLR22b play positive and negative roles in the IFN and NF-κB pathways, respectively. Furthermore, IRAK3, a negative regulator in the MyD88-dependent pathway, was suppressed by CiTLR22a and enhanced by CiTLR22b under poly(I:C) stimulation. In addition, the promoter activities of IRF3 and IRF7 were also examined, which mediate IFN production. We found that the promoter activity of IRF7 but not IRF3 was markedly up-regulated in the CiTLR22a overexpressed cells, but the promoter activities of both IRF7 and IRF3 were significantly decreased in the cells overexpressing CiTLR22b. These results indicated that CiTLR22a regulates IFN response via IRF7, not IRF3, and CiTLR22b via both IRF7 and IRF3. To verify these results, we performed WB at 24 h post-stimulation with poly(I:C). There was a weak blotting band above the corresponding target band, which was previously confirmed as the phosphorylation form of IRF3 or IRF7 (29). The results indicated that CiTLR22a boosts the protein and phosphorylation levels of IRF7, while CiTLR22b inhibits IRF7 phosphorylation and IRF3 protein levels (Figure 6).

### CiTLR22a Inhibits but CiTLR22b Facilitates GCRV Propagation in CIK Cells

**Figure 7:**
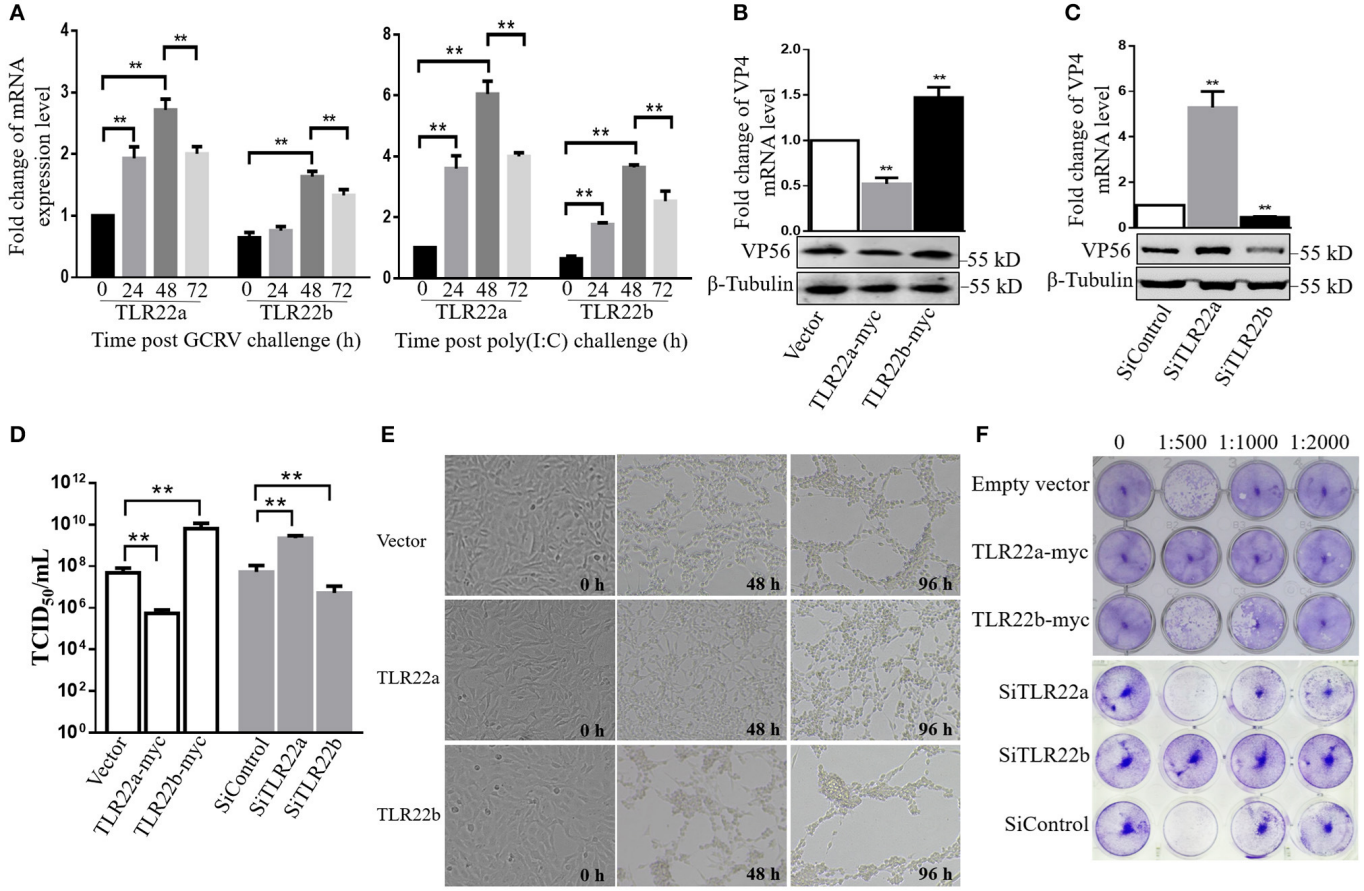
CiTLR22a restricts grass carp reovirus (GCRV) proliferation, while CiTLR22b promotes GCRV replication in CIK cells by qRT-PCR, WB, titer assay, cytopathic effect (CPE), and standard plaque assay. **(A)** mRNA expressions of TLR22a and TLR22b post-GCRV and -poly(I:C) challenge, detected by qRT-PCR. **(B,C)** GCRV quantification in CiTLR22a, CiTLR22b, or empty vector overexpression and interference samples, respectively. The VP4 (major outer capsid protein) expression and VP56 (fiber protein) level were determined at 48 h post-GCRV infection by qRT-PCR (upper panel) and WB (lower panel), respectively. **(D)** Cells were infected with GCRV, and the supernatants were collected at 24 h for viral titer assays by TCID_50_ (50% tissue culture infective dose). **(E)** CPE analysis. The morphology and CPE of TLR22a, TLR22b, and vector cell groups was recorded at 0, 48, and 96 h post-GCRV challenge. **(F)** Standard plaque assay. CIK cells were plated in 24-well plates for 12 h and treated with titer over expression and interference samples of CiTLR22a-myc, CiTLR22b-myc, or empty vector at different dilution rates (0, 1:500, 1:1,000, and 1:2,000). Forty-eight hours later, the viable cells were fixed with 10% paraformaldehyde and stained with 0.05% (w/v) crystal violet. All the experiments were repeated in triplicate. **p < 0.01.

Given the regulation of CiTLR22a and CiTLR22b in IFN and NF-κB production, the roles of CiTLR22a and CiTLR22b in host defense against GCRV infection were investigated. The results showed that the mRNA expression levels of CiTLR22a and CiTLR22b were significantly upregulated upon GCRV and poly(I:C) stimulation (Figure 7A). To confirm the positive and negative roles of CiTLR22a and CiTLR22b in GCRV infection, knockdown and overexpression of CiTLR22a and CiTLR22b were performed. Among the three candidate siRNA sequences for CiTLR22a and the three candidate siRNA sequences for CiTLR22b, s1 for CiTLR22a, and s3 for CiTLR22b showed the highest interference efficiency (Supplementary Figure 2). GCRV was added to the cell culture medium after overexpression or knockdown of CiTLR22a and CiTLR22b. The results showed that the mRNA expression of VP4 (major outer capsid protein) and protein level of VP56 (fiber protein) of GCRV decrease markedly in CiTLR22a overexpression cells and increase significantly in CiTLR22b overexpression cells (Figures 7B,C). GCRV titer significantly reduces in CiTLR22a-overexpressing cells and markedly increases in CiTLR22b-overexpressing cells (Figure 7D). In addition, CPE and standard plaque assays showed that CiTLR22a overexpression significantly promotes cell viability, whereas CiTLR22b overexpression has the opposite effect (Figures 7E,F). In addition, the results above were verified in CiTLR22a- and CiTLR22b-interference cells. These results indicated that CiTLR22a and CiTLR22b play opposite roles in anti-GCRV immune responses.

## Discussion

Earlier study has reported that fugu TLR22 localizes on the cell surface, responds to dsRNA, and recruits TICAM-1 as an adaptor to induce IFN and protect cells from birnaviruses (13). Here, we demonstrated completely distinct signaling pathways of TLR22a and TLR22b in grass carp, which is thoroughly characterized in poikilothermic vertebrates. Our results showed that both CiTLR22a and CiTLR22b localize to lysosome, recruit adaptor molecule MyD88, facilitate or inhibit the phosphorylation level of IRF7, and regulate the IFN and NF-κB pathways. Moreover, we demonstrated that CiTLR22a inhibits the proliferation of GCRV and plays a positive role in antiviral immune responses, while CiTLR22b promotes the proliferation of GCRV and plays a negative role in antiviral immune responses.

Since fugu TLR22 was characterized in 2008 (13), the functions of TLR22 in many other species have been reported, including Asian seabass (*Lates calcarifer*) (45), mudskipper (*Boleophthalmus pectinirostris*) (46), Dabry's sturgeon (*Acipenser dabryanus*) (47), amphioxus (*Branchiostoma lanceolatum*) (48), etc. In all the previous studies, TLR22 responded to dsRNA. In the present study, to explore whether CiTLR22a and CiTLR22b directly interact with poly(I:C), we performed the PAMP-binding and poly(I:C) pull-down assays. The results indicated that poly(I:C) binds CiTLR22a and CiTLR22b in acidic compartment, which is consistent with the result that CiTLR22a and CiTLR22b localize to lysosome. The situation is also in line with human TLR10, which requires acidic pH to bind dsRNA in endosome (42). Although we confirmed that CiTLR22a and CiTLR22b are able to combine poly(I:C), there are still many unsolved issues, for example: What are the specific recognition sites? Is it structurally complementary or affected by charge? Previous research predicted that mutations at p.L159F and p.L529P in the LRR region affect the binding affinity significantly (16). Although there is no direct evidence, it serves as a guide for later research.

As for the subcellular localization of CiTLR22a and CiTLR22b, we found that both of them localize to lysosome, which is thoroughly different from fugu TLR22 located on cell surface (13). In this study, we selected CIK cells to explore the subcellular localization of CiTLR22a and CiTLR22b (same species cells), while fugu TLR22 was mapped to HELA cells (mammalian cells). Different cell types may be the reason for their different subcellular localization. In fact, this hypothesis has been confirmed in human TLR3. In human fibroblasts, TLR3 localizes on the cell surface and inside the cells (49). In contrast, TLR3 expresses inside the cells in monocyte-derived immature DCs and CD11c^+^ blood DCs (50). In addition, temperature might be another reason for the different subcellular localization. Whether grass carp or fugu, the optimal growth temperature is lower than 28°C. Teleost TLR22 protein in the 37°C expressing system may change the protein property and further affect its localization. Moreover, CiTLR22a and CiTLR22b in lysosomes can efficiently sense dsRNA. Previous studies reported that GCRV enters cells by caveolae/raft-mediated endocytosis and induces autophagy (30, 51). During autophagy, autophagosomes encapsulate GCRV and send them to lysosomes for degradation; thus, viral nucleic acids are fully exposed for binding CiTLR22a and CiTLR22b.

MyD88 was identified as an adaptor molecule of CiTLR22a and CiTLR22b. Actually, a previous study indicated that MyD88 is involved in antiviral immunity in grass carp (52). In addition, in our previous research, teleost TLR19 recognizes dsRNA in early endosome and recruits TRIF to initiate downstream signaling pathways (9). Thus, signaling pathways sensing dsRNA initiated by TLRs are not only in early endosome but also in lysosome, not merely through TRIF but also through MyD88, thereby better protecting an animal from pathogen invasion. The results showed that teleosts have a sophisticated and efficient innate immune defense system. In addition, CiTLR22a and CiTLR22b are more dependent on IRF7, but TLR19 is more dependent on IRF3. Human TLR10 recognizes dsRNA in endosome, recruits MyD88, and suppresses IRF7-dependent IFN-I production (42). By analyzing the known TLR pathway, it was found that almost all TLRs recruiting MyD88 are more dependent on IRF7, while TLRs recruiting TRIF are more dependent on IRF3.

In the present study, CiTLR22a and CiTLR22b have the similar signaling pathway. What are the differences between them? To distinguish the functions of CiTLR22a and CiTLR22b, the promotor activities of IFNs, NF-κBs, IRF3/7, and protein and phosphorylation levels of IRF3/7 were investigated. The results showed that CiTLR22a facilitates activations of IRF7, IFNs, and NF-κBs, while CiTLR22b inhibits activations of IRF3/7, IFNs, and NF-κBs. In mammals, IRAK3 negatively regulates TLRs by preventing the dissociation of IRAK and IRAK4 from MyD88 and the formation of IRAK-TRAF6 complexes (10). Correspondingly, the negative regulation of IRAK3 is also proved in grass carp (53). In the present study, we found that CiTLR22a suppresses the expression of IRAK3, while CiTLR22b induces the expression of IRAK3 under poly(I:C) stimulation, which indicated that TLR22a and TLR22b regulate IRAK3 expression upon dsRNA stimulation. In short, CiTLR22a and CiTLR22b show antagonistic functions. The activation of the NF-κB pathway initiates inflammation and facilitates the production of proinflammation cytokines. Although the inflammatory response is critical to control the growth of pathogenic microorganisms, excessive cytokine is harmful to host and can even be fatal. Thus, CiTLR22b, serving as an anti-inflammation TLR, inhibits the release of poly(I:C)-induced excessive cytokines, which is essential for maintaining cellular homeostasis. To further confirm the antagonism in practice, we assessed the ability of resisting GCRV. Compared with the control, CiTLR22a obviously suppresses GCRV proliferation, whereas CiTLR22b promotes GCRV replication. These results indicated that CiTLR22a plays a positive role in antiviral immune responses, whereas CiTLR22b shows the opposite effect in viral infection.

**Figure 8:**
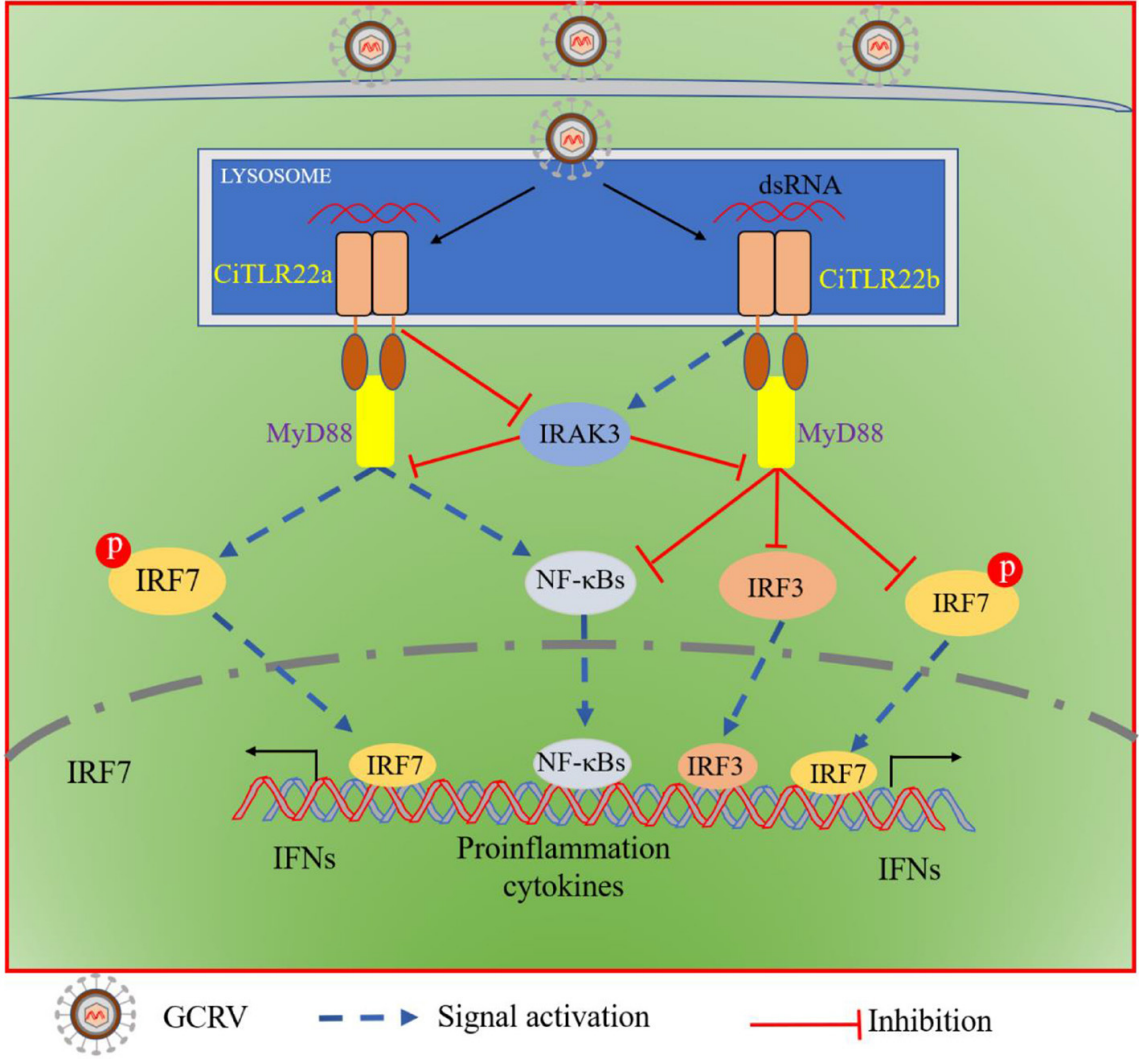
Schematic comparison between CiTLR22a- and CiTLR22b-mediated signaling pathways. Both CiTLR22a and CiTLR22b localize to lysosome, recognize dsRNA, and recruit MyD88 adaptor molecule. CiTLR22a inhibits IRAK3 expression, enhances the protein and phosphorylation levels of IRF7, and facilitates the IFN and NF-κB pathways. However, CiTLR22b promotes IRAK3 expression, inhibits the phosphorylation level of IRF7 and protein level of IRF3, and plays a negative role in initiating the IFN and NF-κB pathways.

In summary, both CiTLR22a and CiTLR22b localize to lysosome, recognize dsRNA, and recruit MyD88 as the adaptor molecule. CiTLR22a enhances protein and phosphorylation levels of IRF7 by suppressing IRAK3 expression, while CiTLR22b inhibits the phosphorylation level of IRF7 and protein level of IRF3 by facilitating IRAK3 expression, and then triggers/suppresses IFN and NF-κB responses (Figure 8). CiTLR22a plays a positive role in antiviral infection, while CiTLR22b shows the opposite effect in antiviral immunity, which maintains the balances of immune responses. The present study systematically clarifies the signaling pathways of two isotype TLR22s; especially, subcellular localization and adaptor molecule are thoroughly different from previous TLR22 research, which results from technical limitations. This research also presents the first direct evidence that TLR22 binds dsRNA at acidic pH condition. The results serve the antiviral immune mechanisms in poikilothermic vertebrates and evolutionary immunology.

## Data Availability Statement

The datasets generated for this study can be found in GenBank, with the following accession numbers. CiTLR22a at HQ676542; CiTLR22b at KY824797; MyD88 at FJ843088; TIRAP at KF735057; TRIF at KC333648; and IRAK-M at MH590729.

## Author Contributions

JSu and JJ conceived and designed the experiments, and wrote the manuscript. JJ, YR, WL, CY, and GY performed the experiments and analyzed the data. ZL and HF contributed to the bioinformatics analysis of correlative gene sequences. JSh and ZX revised the manuscript critically. All authors reviewed the manuscript.

### Conflict of Interest

The authors declare that the research was conducted in the absence of any commercial or financial relationships that could be construed as a potential conflict of interest.

## Abbreviations

BCAP: B-cell adaptor for PI3K
CIK: *Ctenopharyngodon idella* kidney cell line
DC: dendritic cell
EF1α: elongation factor 1α
eGFP: enhanced GFP
GCRV: grass carp reovirus
IB: immunoblot
IP: immunoprecipitation
IRAK: IL-1 receptor-associated kinase
IRF: IFN regulatory factor
LRR: leucine-rich repeat motif
PAMP: pathogen-associated molecular pattern
poly(I:C): polyinosinic:polycytidylic acid
RFP: red fluorescent protein
RAB: ras related in brain
SARM1: sterile alpha and armadillo-motif containing protein 1
TICAM-1: TIR domain-containing adapter molecule 1
TIR: Toll/interleukin-1 receptor
TIRAP: TIR domain-containing adaptor protein
TRAF6: TNF receptor-associated factor 6
TRIF: TIR-domain-containing adapter-inducing interferon-β
WB: Western blotting.
