# Supplementary material for: Thoroughly Remold the Localization and Signaling Pathway of TLR22
Source: Front Immunol. 2020 Jan 17;10:3003. doi: 10.3389/fimmu.2019.03003 (PMC6978911; doi:10.3389/fimmu.2019.03003)
Supplement: Supplementary file 1 [file Table_1.docx]

**Supplementary Table 1** Primers for vector constructions.

| Primer | Sequence (5’-3’) | Application |
| --- | --- | --- |
| TaF271  TaR272  TaF122  TaR123  129  130  TbF273  TbR274  TbF124  TbR125  131  132  TpF364 | GGggtaccAATCAAGGCAAATCCAATAGA  ATCGgggcccTCACAGATCCTCTTCAGAGATGAGTTTCTGCTCAATTGCACTCTCTGGGTTA  GGggtaccAATCAAGGCAAATCCAATAGA  CGggatccATTGCACTCTCTGGGTTATGA  CGCggatccAAAAAAGAATCAAGAAAAAGC  CCGctcgagTTAGGTGCAGGATTCGATGTTGAA  GgaattcCTGGATGATTTACACCATTTAAGT  ATCGgggcccTCACAGATCCTCTTCAGAGATGAGTTTCTGCTCCCCCAAAATCCCAGCCAAT  ACTGgggcccAATGTCGAAGAGAGAGGCTGGT  CGgaattcATCCCAGCCAATGGATGCTC  CCGgaattcTACTCAGTAAAGAACTGTACCGTCAG  CCGctcgagTTAGTTACATGAATCTACATCAAGATCCAG  ACTGgaattc GAATGGAGGAAAACCGCGT | TLR22a-myc  TLR22a-eGFP  LRR-TLR22a  TLR22b-myc  TLR22b-eGFP  LRR-TLR22b  TIRAP-HA |
| TpR365  TF-F366  TF-R367  MDF401  MDR402 | GGggtaccTCAAGCGTAGTCTGGGACGTCGTATGGGTAGCTGTCTGACTGAGACGCATT GGggtaccATGGCAGAAGAGCTTATGGA  CGggatccAGCGTAGTCTGGGACGTCGTATGGGTACATAAAGTCAAACTCATCAGCTG  GGggtaccTCACTTATCGTCGTCATCCTTGTAATCTTTTTGTTAGTTGGGAAAGG  CGgaattcGCTGGTAGATGTTTTTGTCC | TRIF-HA  HA-MyD88 |

Notes: The nucleotides in lower case stand for the site of restricted enzyme. The upper case letters in front of lower case represent protective bases.
