# Supplementary material for: Thoroughly Remold the Localization and Signaling Pathway of TLR22
Source: Front Immunol. 2020 Jan 17;10:3003. doi: 10.3389/fimmu.2019.03003 (PMC6978911; doi:10.3389/fimmu.2019.03003)
Supplement: Supplementary file 2 [file Table_2.docx]

# Supplementary Table 2 Primers for qRT-PCR analysis.

| Gene | Primer name | Forward primer (5'–3') | Primer name | Reverse primer (5'–3') |
| --- | --- | --- | --- | --- |
| EF1α | EF125 | CGCCAGTGTTGCCTTCGT | ER126 | CGCTCAATCTTCCATCCCTT |
| IRAK3 | IKF166 | GCTTTAGCTGCTCGCATCCTTC | IKR167 | CATTATTTGGCAGATTCGTGGC |
| VP4 | VF146 | CGAAAACCTACCAGTGGATAATG | VR147 | CCAGCTAATACGCCAACGAC |
