# Supplementary material for: Thoroughly Remold the Localization and Signaling Pathway of TLR22
Source: Front Immunol. 2020 Jan 17;10:3003. doi: 10.3389/fimmu.2019.03003 (PMC6978911; doi:10.3389/fimmu.2019.03003)
Supplement: Supplementary file 3 [file Image_1.pdf]

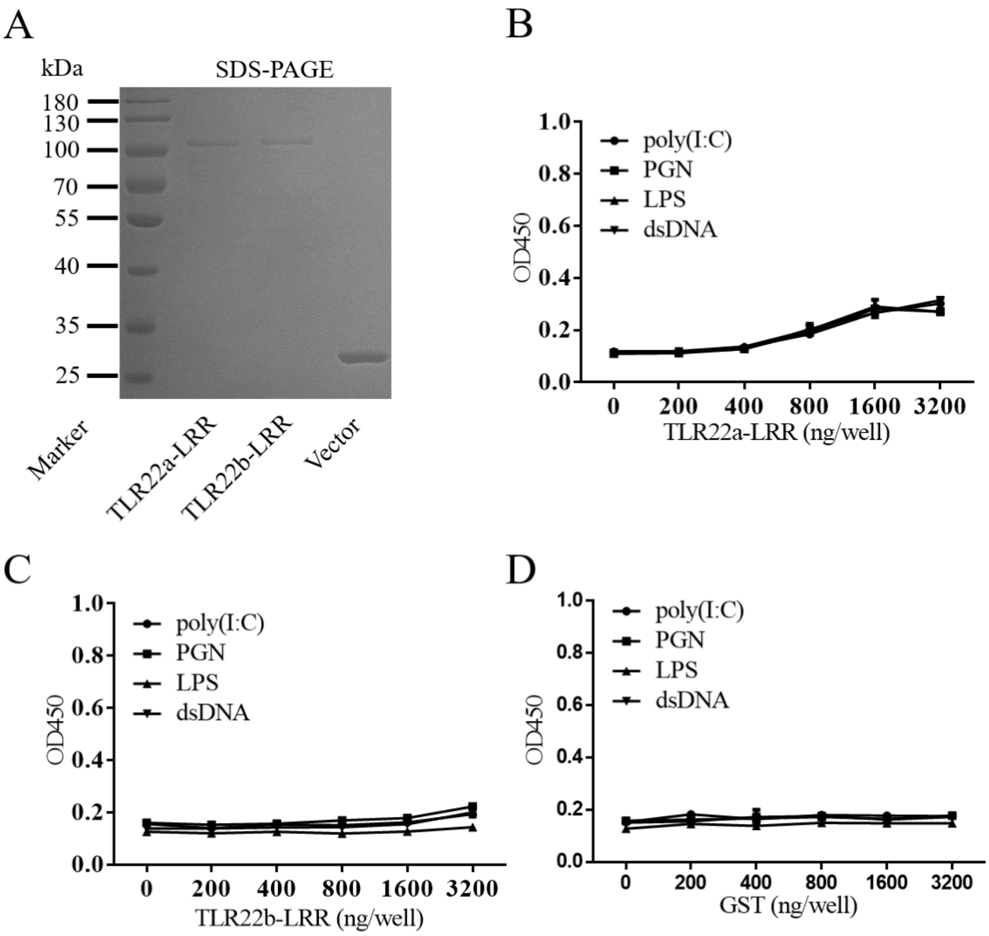

Supplementary Figure 1 The interaction between CiTLR22a-LRR, CiTLR22b-LRR and PAMPs were not detectable *in vitro*(A) Purified CiTLR22a-LRR and CiTLR22b-LRR recombinant proteins were analyzed by SDS-PAGE. (B-D) The binding abilities between CiTLR22a-LRR, CiTLR22b-LRR, GST and different PAMPs were analyzed by ELISA at pH 7.4. The results are representative of the mean ± SD (n=4).
