# Supplementary figures and images for: Thoroughly Remold the Localization and Signaling Pathway of TLR22
Source: Front Immunol. 2020 Jan 17;10:3003. doi: 10.3389/fimmu.2019.03003 (PMC6978911; doi:10.3389/fimmu.2019.03003)

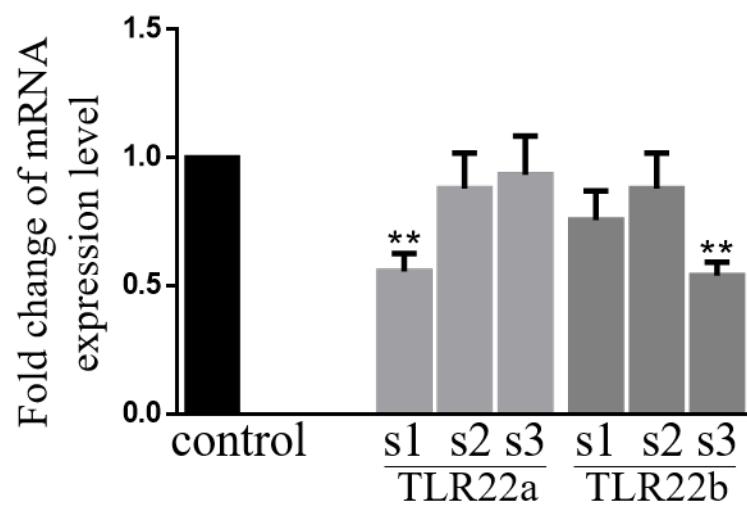

**Supplementary Figure 2:** Efficiency of TLR22a and TLR22b RNAi, detected by qRT-PCR.

Supplement: Supplementary file 4 [file Image_2.pdf]
